# Effect of Physical Properties on Mechanical Behaviors of Sandstone under Uniaxial and Triaxial Compressions

**DOI:** 10.3390/ma16134867

**Published:** 2023-07-06

**Authors:** Esraa M. Alomari, Kam W. Ng, Lokendra Khatri, Shaun S. Wulff

**Affiliations:** 1 Department of Civil and Architectural Engineering and Construction Management, University of Wyoming, Laramie, WY 82071, USA; ealomari@uwyo.edu (E.M.A.); lokendra.khatri320@gmail.com (L.K.); 2 Department of Mathematics and Statistics, University of Wyoming, Laramie, WY 82071, USA; wulff@uwyo.edu

**Keywords:** compressive strength, confining stress, porosity, water content, young’s modulus

## Abstract

Mechanical properties of sandstone, such as compressive strength and young’s modulus, are commonly used in the design of geotechnical structures and numerical simulation of underground reservoirs using models such as the digital groundwater, equivalent porous medium, and Discrete Fracture Network (DFN) models. A better understanding of the mechanical behaviors of sandstone under different loading conditions is imperative when assessing the stability of geotechnical structures. This paper highlights the effect of the physical properties (i.e., porosity, mean grain size) and environmental conditions (i.e., water content and confining stress) on uniaxial compressive strength, triaxial compressive strength, and young’s modulus of sandstone. A series of uniaxial and triaxial compression experiments are conducted on sandstone formations from Wyoming. In addition, experimental data on sandstones from the literature are compiled and integrated into this study. Prediction equations for the compressive strengths and young’s modulus of sandstone are established based on commonly available physical properties and known environmental conditions. The results show that the mean Uniaxial Compressive Strength (UCS) decreases as the porosity, water content, and mean grain size increase. Furthermore, a predictive empirical relationship for the triaxial compressive strength is established under different confinements and porosity. The relationship suggests that the mean peak compressive strength increases at a higher confinement and decreases at a higher porosity. The results and recommendations provide a useful framework for evaluating the strength and deformation of most sandstone.

## 1. Introduction

Sandstone is a common sedimentary rock that is widely distributed on the crust surface of the earth [1]. Sandstone is considered a major reservoir system that can be an oil- and gas-rich depositional basin such as the Permian Shanxi sandstone on the southern edge of the Yimeng uplift in China [2]. Also, sandstones can be major aquifers for groundwater flow and contaminants transportation [3]. An understanding of the strength and deformation behaviors of sandstone is essential for the design and simulation of geotechnical structures for underground environments and underground reservoirs for mineral extraction and subsurface storage [4]. Although the mechanical behaviors of sandstone have been investigated in past studies, a more comprehensive study that involves a wide variety of sandstone formations under different loading conditions is important to understand sandstone behaviors and application in engineering designs and constructions. This study will investigate the uniaxial compressive strength (i.e., the compressive strength under zero confinement) and the triaxial compressive strength (under different confinements), followed by the deformation behavior of sandstone based on Young’s modulus.

The uniaxial compressive strength (UCS) of sandstone can be affected by several factors like water content, pore space, and mean size of grain particles. The water weakening effect can be problematic for the stability of rock structures due to interactions between water and silicate minerals. Water weakens the hydrogen bonds, promotes the dissolution of clay minerals, and creates voids that can change the rock microstructure and ultimately weakens the rock [5,6,7,8,9,10]. In addition, pores inside a rock matrix act as flaws that create stress concentration and lead to macroscopic failure [11]. Therefore, a rock with more porous microstructures has more voids that can reduce the strength and stiffness of the rock matrix [12,13,14,15]. However, past studies considered the individual effect of water content and porosity while their coupled effect on the mechanical behavior of sandstone is rarely understood even though sandstone is typically situated in a moist or saturated environment rather than a dry condition. In addition, the UCS of sandstone tends to decrease as the mean grain size increases [16,17].

The triaxial compressive strength of sandstone is affected by several factors, such as water content, confining stress, and porosity. Triaxial compression test results revealed a positive effect of confinement on compressive strength [1,7,18,19,20]. The influence of confining stress on the triaxial compressive strength of sandstone has been a subject of earlier investigations. However, the effect of rock porosity on the triaxial compressive strength of sandstone under different confinements is rarely understood and deserves further investigation.

This paper presents the major factors influencing the uniaxial and triaxial compressive strengths and Young’s modulus. A study is completed to achieve the following objectives:

i. Systematically investigate the influence of physical and environmental factors on the mechanical and deformation behaviors of sandstone;
ii. Developing empirical models to predict the compressive strength of sandstone based upon commonly measured properties such as water content, porosity, mean grain size, and confining stress;
iii. Providing valuable findings to improve the understanding and prediction of mechanical behaviors of sandstone that will help to increase the safety and the cost-effectiveness of the built environment and structures supported by sandstone.

## 2. Regression Modeling

Linear and nonlinear models are used to examine the relationship between a response variable $Y_{i}$ and an observed vector of predictors $x_{i}\;$ for observation i as follows:

(1) $$Y_{i}=f\left(x_{i},\;\beta \right)+E_{i}$$

where β is a vector of regression coefficients, $E_{i}$ is a random error term, and f is a linear or nonlinear function with respect to β. Thus, the model in (1) involves the specification of f and the choice of predictors $x_{i}$.

A dataset is split randomly into a training dataset and a testing dataset [21]. The training set is used to fit and select a model. The testing set is used to evaluate the predictive ability of the model. However, it is important that the training set is sufficiently large for an adequate model to be developed. The selected model in Equation (1) is fit to the training data to obtain the estimated vector of regression coefficients $\left(\hat{\beta }\right)$. This fitted model is then used to generate the predicted values of the response variable $\left({\hat{y}}_{i}\right)$ as follows.

(2) $${\hat{y}}_{\left(i\right)}=f\left(x_{\left(i\right)},\;\hat{\beta }\right)$$

The selected model can be evaluated by comparing the observed values of the response variable $\left(y_{i}\right)$ to the predicted values of the response variable $\left({\hat{y}}_{i}\right)$. Two commonly used measures for this comparison are the Root Mean Square Error (RMSE) given by Equation (3) and the Mean Absolute Deviation (MAD) given by Equation (4):

(3) $$\mathrm{RMSE}=\sqrt{\frac{\sum {\left(y_{\left(i\right)}-{\hat{y}}_{\left(i\right)}\right)}^{2}}{num}}$$

(4) $$\mathrm{MAD}=\frac{\sum \left|y_{\left(i\right)}-{\hat{y}}_{\left(i\right)}\right|}{num}$$

where *num* is the number of observations. It is desirable to have a small RMSE and MAD for a reasonable candidate model. Predictions are generated for both the training dataset and the testing dataset. Predictions based on the training dataset are used to assess the goodness-of-fit of a set of initially proposed models. Predictions based upon the testing dataset are used to assess the predictive ability of models proposed by this research and the models appearing in the literature.

When there are multiple predictors, the choice of the predictors in Equation (1) can be difficult since these predictors could have relationships with the true mean of the response that is linear, nonlinear, or some combination of both. Thus, the relationships between the response and the predictors are examined visually using scatterplots. The scatterplots are enhanced by adding a locally weighted polynomial regression fit to emphasize the relationship [22]. Additive regression models are also fit to statistically assess linear and nonlinear relationships between the true mean of the response and the collection of available predictors. The additive regression model (GAM) has the form of Equation (1) where $f\left(x_{i},\;\beta \right)$ is replaced by

(5) $$f\left(x_{i}\right)=\alpha +s_{1}\left(x_{1i}\right)++\cdots +s_{p}\left(x_{pi}\right)$$

where $x_{ki}\;$ denotes predictor variable k, α denotes the intercept, $\;s_{k}$ denotes the corresponding smoothing spline for predictor variable k, and k=1,2,…,p [23]. The additive models are fitted using the GAM function in the GAM package available in the R Program [24]. Hypothesis tests are conducted on the linear trend and the nonlinear trend associated with $x_{ki}$ on the true mean of the response $Y_{i}$.

## 3. Unconfined Compressive Strength (UCS)

### 3.1. Sample Preparation and Test Equipment

An experimental study is carried out on 13 sandstone formations from Wyoming, USA, to examine the effect of physical properties and confining stress on mechanical behaviors. Rotary drilling of rock cores and boulders is used to extract testing specimens. The rock specimen length is cut to obtain a length-to-diameter ratio (L/D) of two according to the ASTM standards, and both ends of the rock specimens are trimmed and polished.

The effect of the specimen size has been investigated by several researchers with most of them focusing on UC tests. A study by Hoek and Brown [25] conducted on specimens with a diameter range from 15 to 200 mm showed that UCS increases progressively as the sample diameter decreases. Other studies, such as Li et al. [26], found that the scale effect proposed by Hoek and Brown (1980) did not hold for sandstone in which there is an increase in UCS with specimen diameter based on a study conducted on specimens with a diameter range of 12.7 to 101.6 mm. Compression tests in this study are conducted on 25- and 50 mm diameter sandstone specimens in addition to other diameters for sandstones collected from the literature that are presented in Table 1, Table 2 and Table 3. The sample size effect is ignored due to the narrow range of test sample diameters ranging from 20 to 50 mm.

**Figure 1:**
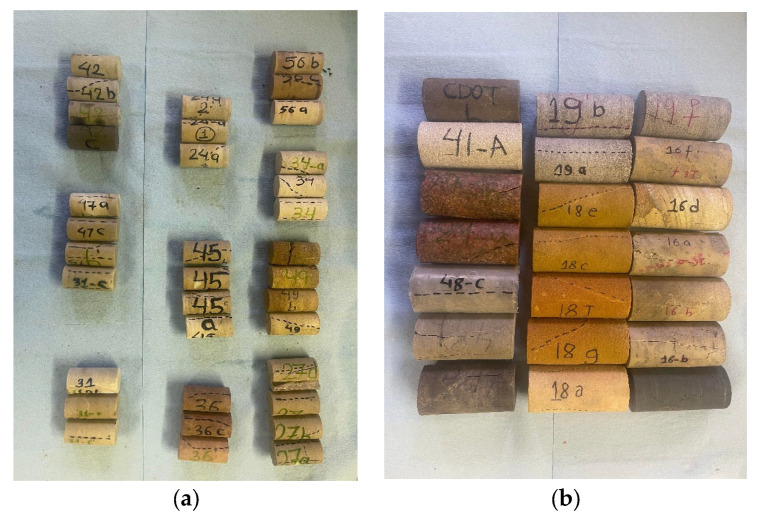
Tested sandstone specimens from Wyoming showing (**a**) 25 mm in diameter and (**b**) 50 mm in diameter.

ASTM recommends using samples of 2:1 (height: diameter) ratio for testing. The advisability of using cores of 2:1 factor is indicated by John (1972) [40]. He demonstrated that for dry sandstone, the compressive strengths obtained are approximately the same for cores of a height/diameter ratio of 2:1 or greater, but for a smaller ratio, there is a notable increase in strength. In this study, all our tested sandstones and the sandstones collected in the literature have a height/diameter ratio of 2:1, as shown in Figure 1.

All specimens are oven-dried for 24 h to assure that they are completely dry before testing. No macroscale defects are observed in any of the specimens before testing. The porosity (*n*) of each sandstone specimen is calculated using the gravimetric method by determining the specific gravity and moisture content. The specific gravity is determined using the AASHTO-100 standard [41] test method. Then, the porosity is calculated by:

(6) $$Porosity\;\left(\%\right)=\frac{1-dry\;bulk\;density}{specific\;gravity}\times 100$$

Average porosity values range from 3.43% to 31.2% with an average dry density of 2.406 g/cm^3^. Mean grain sizes are examined using a glass magnifier with high-power magnification from which the grain size is estimated based on the size scale of sandstone. The data of grain size obtained from the size scale of the magnifier are subdivided into three categories: fine, medium, and coarse-grained according to the USGS classification scheme for sandstone [42].

**Figure 2:**
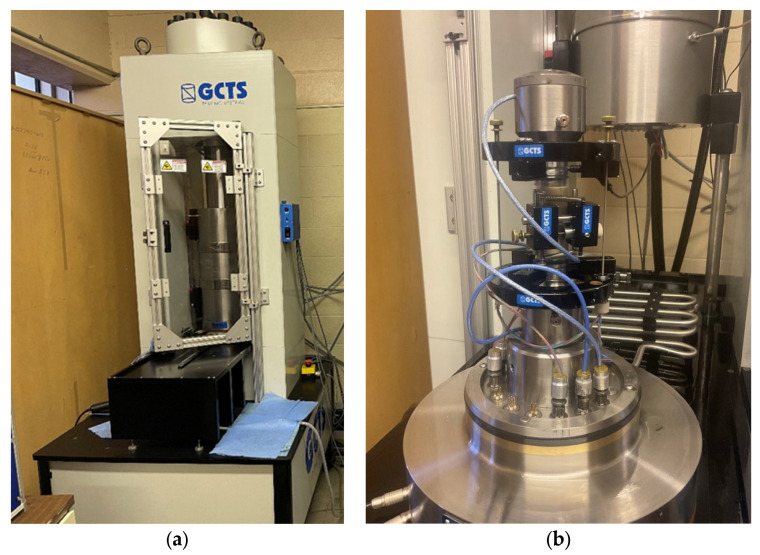
(**a**) GCTS RTR-1500 uniaxial and triaxial testing equipment, (**b**) Triaxial setup with three LVDT sensors.

The UC and triaxial tests are conducted at room temperature using the testing system (GCTS RTR-1500) at the University of Wyoming in accordance with ASTM D7012 (2014) [43], as shown in Figure 2a. This loading system has a maximum compression load of 1500 kN, and a tension load of 818 kN and can test most rock types using a controlled axial strain setup at a constant strain rate of 0.1% per minute for hard rocks and 0.05% per minute for softer rocks. Each rock specimen is instrumented with three Linear Variable Differential Transformers (LVDTs) to measure two axial strains and a radial strain, as shown in Figure 2b. The load and deformation responses of each test are recorded simultaneously at a data collection interval of 0.5 s.

Young’s modulus was determined based on axial stress–strain data using a moving average regression analysis method [44]. A linear part of the stress–strain plot was determined for a range of stress–strain data that yields the highest coefficient of determination (R^2^) closer to 1. Young’s modulus was calculated as the slope or gradient of the best linear stress–strain curve.

### 3.2. Uniaxial Compression Testing

A total of twelve sandstone samples of different formations and porosities are tested under a UC condition and at room temperature. A summary of the UC test results is given in Table 1.

Rocks with higher porosities tend to have lower UCS and *E* values. For example, Flathead Sandstone with a porosity of 3.06% exhibits higher UCS and *E* values by 43% and 66%, respectively, than that of Cloverly Sandstone with a porosity of 21.2%.

### 3.3. Historical Sandstone Data from WYDOT Database

Additional test results of four different sandstone formations are collected from a historical rock database developed by the Wyoming Department of Transportation (WYDOT). The laboratory tests were conducted in the WYDOT’s certified laboratory and conducted by trained laboratory engineers. These sandstones are tested under different water contents and porosities. Table 2 summarizes 36 UC test results of the four sandstone formations.

Sandstones with the lowest water content and porosity have the highest UCS values. For example, Aspen Sandstone with the lowest water content ranging from 0.51 to 3.19% and a relatively low porosity of 3.4% exhibits the highest UCS ranging from 11.43 to 161.34 MPa.

### 3.4. Experimental Sandstone Data from the Literature

Experimental data compiled from the literature are utilized to better understand the mechanical behaviors of sandstone from different regions of the world. Sixty-one sandstone formations collected from the literature, in addition to sixteen formations from Wyoming, USA, were tested under UC. However, other sandstone formations such as Bentheim and Fontainebleau sandstones were not included in this study due to relatively different testing conditions such as specimen shape (i.e., prismatic), drainage condition during compression (i.e., deformation is fully drained), and principal stresses condition (i.e., biaxial) [45,46]. Sandstone formations and properties are summarized in Table 3.

### 3.5. Relationship between UCS, Water Content, Mean Grain Size and Porosity

**Figure 3:**
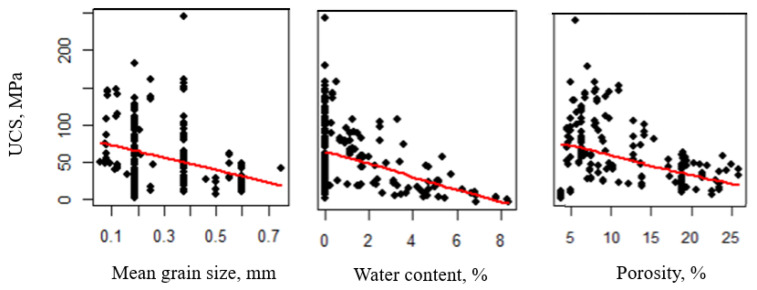
Linear trend associated with the predictor variables on the true mean of the response UCS.

UCS is one of the most commonly measured rock parameters in rock engineering [47]. Compressive strength generally decreases with the increase in water content, porosity, and mean grain size. Various models are evaluated to the mean UCS based on the predictor’s mean grain size, porosity, and water content after the exclusion of one outlier. The GAM is only able to provide marginal evidence against claims of linear trends in the predictors on the true mean UCS, as shown in Figure 3 where the black squares represent experimental data points of the training dataset, and the red lines represent the trend of the response variable and the predictors. While it might be possible to identify complex nonlinear trends in these predictors, such models are not pursued here for the sake of simplicity and to prevent overfitting. Thus, the recommended model for the true mean UCS is linear in the predictors mean grain size ($d_{m}$ in mm), porosity (*n* in %), and water content (*w* in %), as shown in Figure 3 and given by Equation (7) based on the training dataset that contains 193 data points with 31 formations from the literature and four formations from Wyoming.

Since some sandstones from the literature, in addition to our tested sandstones, only have size classification and no exact grain size, the USGS classification scheme for sandstone [42] assumes a specific grain diameter for each size classification (i.e., for fine sandstone, the upper and lower grain diameter is 250 and 125 μm, respectively). Several data points are clustered at the same grain size and have different UCS values since they have the same size classification but different water contents and porosities. Also, some sandstones are tested under dry condition, which explains the large amount of data at *w* = 0 in the scatterplot of UCS versus *w* (Figure 3).

(7) $$\hat{\mathrm{UCS}}\;\left(MPa\right)=108.46-2.23\;n-8.78\;w-38.77\;d_{m}$$

It is desirable to test porous rocks such as sandstone under moist and saturated conditions so that a comparison can be made under dry and saturated levels, and an estimate for the lower bound of design parameters can be used [48]. The negative linear trend in water content on predicting UCS from Equation (7) is consistent with past findings that explain the water weakening effect as a combination of mechanical and chemical processes that occur at a microscopic scale [49].

The water weakening effect includes the dissolution of cement inside the rock that leads to the loosening of the internal microstructure [6]. Furthermore, the increase in water saturation means more voids are occupied with water and increases the likelihood of slippage between solid particles. For example, the UCS of Shanxi Sandstone in Table 3 with a porosity of 7.90% decreases from 66.45 to 40.62 MPa or 39% when the water content increases from 0 to 2.96%, emphasizing the weakening effect of saturation [33]. Similarly, an increase in porosity reduces the predicted UCS values of both dry and saturated sandstones because pores are considered weak points within a rock matrix that induces stress concentration. Hence, more porous sandstones have more voids and higher porosity, reducing the strength of the rock skeleton [15]. The negative linear trend of the mean grain size on the predicted UCS from Equation (7) is consistent with the past findings on artificial sandstones [50]. This observation can be explained by the fact that larger grains have longer grain boundaries, which provide more flaws for the nucleation of cracks and stress concentration [51].

Table 4 summarizes several relationships for predicting the UCS of specific sandstone formations reported in the literature.

These relationships are developed based on a single predictor variable of either water content (*w*) or porosity (*n*) in percentage. According to the independent testing dataset that contains 78 data points of 19 sandstone formations from the literature and four sandstone formations from Wyoming, RMSE values ranging from 31.6 to 58.31 and MAD values ranging from 22.13 to 45.55 are calculated for the comparison of the different equations. The proposed Equation (7) for UCS prediction fits the testing dataset better than other equations according to the lowest RMSE of 31.6 and MAD of 22.13 summarized in Table 4.

## 4. Triaxial Compressive Strength

### 4.1. Conventional Triaxial Compression Testing

Conventional triaxial compression tests are conducted on 17 samples from 13 sandstone formations. GCTS RTR-1500 equipment has rapid, easy, and safe operation with automated cell assembly and meets the specifications of the ISRM and ASTM standards for triaxial testing of the rock samples. The axial load actuator has a capacity ranging up to 1500 kN and the triaxial cell can apply a maximum confining pressure of 140 MPa. The confinement is applied using an oil-filled stainless-steel chamber inside the frame. The initial seating pressure of 0.345 MPa is applied before the shearing stage. A summary of the triaxial compression test results is given in Table 5.

### 4.2. Experimental Sandstone Data from the Literature

Thirty sandstone formations collected from the literature and thirteen formations from Wyoming, USA, were tested under triaxial compression under confining stresses ranging from 1 to 90 MPa. These formations, with a porosity of 1.5 to 21%, are summarized in Table 6.

### 4.3. Effect of Porosity and Confining Stress

**Figure 4:**
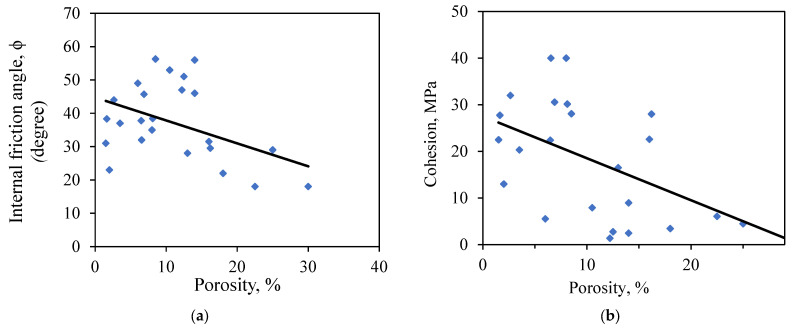
Comparison of the internal friction angle and porosity based on the training dataset.

The effect of porosity on rock strength is further demonstrated with a comparison of porosity and the failure parameters, cohesion (c), and internal friction angle (ϕ) seperately, derived for the Mohr–Coulomb criterion as shown in Figure 4. Figure 4a shows the relationship between porosity and the internal friction angle, whereas Figure 4b shows the relationship between porosity and cohesion. The blue squares represent experimental data of cohesion and internal friction angle resulting from triaxial compression tests of tested sandstone fitted by linear black lines to check the significance of porosity on the response variables (i.e., c and ϕ).

The ϕ decreases with increasing porosity indicating that the porosity has a significant effect on the rock failure, as shown in Figure 4a. A similar decreasing trend is observed between porosity and cohesion. However, since cohesion depends on other factors that are not available in this study, such as mineralogy, geological process (cementation), and compaction, a weak relationship and large variation is observed between porosity and cohesion, as shown in Figure 4b, compared to the relationship between the internal friction angle and porosity.

The peak compressive strength $\sigma_{1}\;$decreases with the increase in n. An increase in the internal surface area per unit rock volume resulting from a higher n decreases the predicted integrity of the rock and hence reduces its strength [16]. Compressive strength decreases as the porosity increases. This observation is explained by the weakening effect of higher porosity on the rock strength since a higher volume of voids induces stress concentrations inside the rock matrix. On the other hand, the mean $\sigma_{1}$ generally increases with an increase in the confining stress $(\sigma_{3})\;$ due to the strengthening effect of confinement on compressive strength.

**Figure 5:**
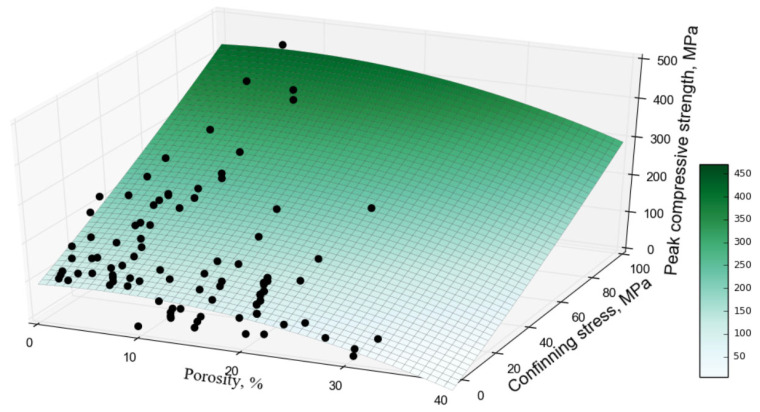
Relationship between the peak compressive strength and the rock porosity in % and confining stress in MPa (Edited).

The test results from the GAM indicate that both n and $\sigma_{3}$ are important predictors of mean $\sigma_{1}$. The GAM test results do not indicate evidence against a simple linear trend of $\sigma_{3}\;$ on the true mean $\sigma_{1}$, but do provide evidence against a simple linear trend of *n* on the true mean $\sigma_{1}$. As a result, the proposed Equation (8) contains a polynomial of order two to capture the nonlinear relationship between *n* in %, $\sigma_{3}\;$in MPa and the true mean $\sigma_{1}$ in MPa based on the training dataset that contains 61 data points represented by the black circles in Figure 5 with 17 sandstone formations from the literature and the 13 sandstone formations from Wyoming, as shown in Figure 5.

(8) $$\hat{\sigma_{1}}=90.68+1.63\;n\%+2.9\;\sigma_{3}-0.12\;n{\%}^{2}+0.0042\;\sigma_{3}^{2}$$

Previously published relationships related $\sigma_{1}$ to $\sigma_{3}$ without considering the effect of porosity. Table 7 summarizes several relationships for predicting the $\sigma_{1}$ of specific sandstone formations reported in the literature.

According to the independent testing dataset that contains 43 data points of 15 sandstone formations from the literature and three sandstone formations from Wyoming, RMSE values ranging from 57.18 to 169.05 and MAD values ranging from 44.40 to 119.59 are calculated for comparing the different equations. The proposed Equation (8) for $\sigma_{1}$ prediction fits the testing dataset better than other equations according to the lowest RMSE of 50.19 and MAD of 40.60 summarized in Table 7.

## 5. Young’s Modulus

**Figure 6:**
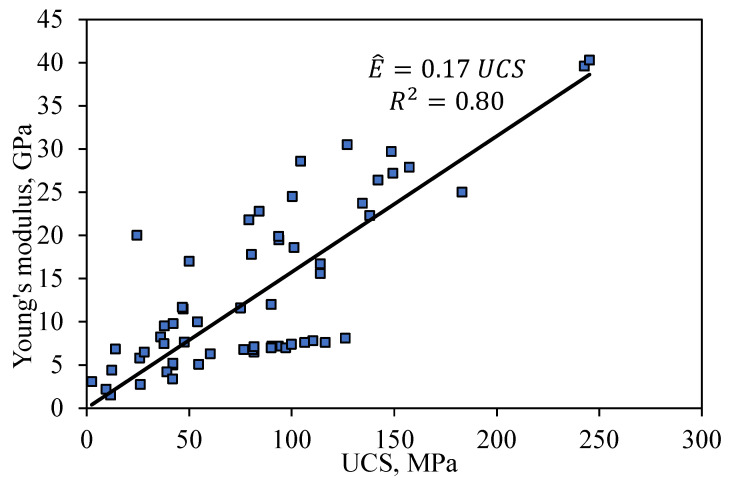
Relationship between UCS and Young’s modulus *E* of the training dataset.

Beside the compressive strength of sandstone, it is important to understand the deformation behavior of sandstone characterized by Young’s modulus. The data presented in Table 1, Table 2 and Table 3 is split randomly into training and testing datasets. The training dataset contains 90 UC data points with 16 sandstone formations from the literature and 12 sandstone formations from Wyoming (mentioned in Table 1), whereas the testing dataset contains 39 data points represented by the black squares in Figure 6 for 13 sandstone formations from the literature and 1 sandstone formation from Wyoming. The relationship between Young’s modulus (*E*) and UCS according to the training dataset is shown in Figure 6 represented by the black linear fit. The plot illustrates a linear increase in mean *E* as UCS increases with a statistical measure R-squared of 0.80.

The prediction equation given by Equation (9) describes a linear increase in the predicted Young’s modulus ($\hat{E}$) with an increase in UCS. Other related studies predicted UCS linearly to *E,* as summarized in Table 8 [12,61,62]

(9) $$\hat{E}\left(GPa\right)=0.17\times \mathrm{UCS}\left(\mathrm{MPa}\right)$$

A comparison of the proposed Equation (9) with other relationships reported in the literature is presented in Table 8.

According to the testing dataset, the proposed Equation (9) has the lowest RMSE of 9.79 and MAD of 7.05 compared to those from the literature, indicating a better prediction of the Young’s modulus.

## 6. Conclusions

A series of uniaxial and triaxial compression tests are carried out in addition to experimental data collected from the literature to study the effects of physical properties and environmental conditions on the mechanical behaviors of sandstone under different loading conditions. The main findings drawn from this study are as follows:

1. The uniaxial compressive strength of both dry and saturated sandstones is linearly related to water content, porosity, and mean grain size.
2. For the triaxial compression condition, the internal friction angle is influenced by porosity. The internal friction angle decreases with the increase in porosity. A similar negative trend is observed between porosity and cohesion.
3. A significant effect of the porosity and confining stress on the triaxial compressive strength was observed on dry sandstones. The results of this study offer insights into how commonly measured properties can be utilized to improve the engineering design of sandstone structures.
4. Particularly, the research findings from this study are established based on different sandstone formations from all over the world. Hence, the proposed empirical equations provide a better prediction of sandstone mechanical properties.

## Figures and Tables

**Table 1:** Summary of the UC test results of sandstone formations in Wyoming.

Sample ID	Formation	Geological Age	Depth, m	Size Classification	D, mm	*n*, %	*w*, %	UCS, MPa	*E*, GPa
16	Flathead	Cambrian	6.04	Medium	50	3.06	0	20.31	2.60
17	Cloverly	Cretaceous	30.34	Medium	50	21.20	0	11.61	1.51
18	Sundance	Jurassic	6.49	Fine	50	23.20	0	13.88	24.08
19	Aspen	Cretaceous	6.19	Medium	50	3.69	0	22.59	NA
23	Lance	Cretaceous	Surface	Fine	50	13.82	0	2.47	3.067
31	Tensleep	Pennsylvanian	Surface	Fine	50	12.80	0	55.90	12.79
32	Arikaree	Lower Miocene	Surface	Coarse	50	10.90	0	12.18	4.39
33					50	12.10	0	17.00	4.44
39	Hanna	Paleogene	43.96	Fine	25	13.80	0	9.39	2.21
41			23.26	Coarse	25	15.40	0	9.65	1.91
43	Wind River	Eocene	Surface	Medium	25	13.60	0	47.59	7.64
49	Bridger	Eocene	Surface	Medium	25	26.20	0	13.91	6.83
50	Fort Union	Paleocene	Surface	Medium	25	13.80	0	6.00	1.15
51					25	3.92	0	26.08	2.75
56	Casper	Permian	Surface	Medium	25	9.37	0	39.00	9.54

D—Specimen diameter in mm, *n*—Porosity in percentage, *w*—water content in percentage, UCS—Uniaxial Compressive Strength in MPa, *E*—Young’s modulus in GPa.

**Table 2:** A summary of UC test data from historical WYDOT database.

Formation	Geological Age	Size Classification	D, mm	*n*, %	Number of UC test	*w*, %	UCS, MPa
Flathead	Cambrian	Fine	50	2.10	12	4.49–8.88	1.86–12.79
Cloverly	Cretaceous	Medium	50	18.00	10	0.27–5.33	10.56–61.21
Sundance	Jurassic	Fine	50	23.00	5	3.44–5.74	14.38–38.62
Aspen	Cretaceous	Medium	50	3.40	9	0.51–3.19	11.43–161.43

D—Specimen diameter in mm, *n*—Porosity in percentage, *w*—water content in percentage, UCS—Uniaxial Compressive Strength in MPa.

**Table 3:** Summary of UC test results of sandstones from the literature.

The Formation, (Location)	Size Classification	D, mm	*n*, %	*w*, %	UCS, MPa	*E*, GPa	Reference
NA, (France, Germany, USA, UK)	Medium	20	6.50–28.00	0	161.40–30.10	NA	Baud et al. (2014) [27]
NA, (Chuxiong)	Fine	50	8.50	0–2.29	71.91–50.48	7.05–5.52	Cai et al. (2019) [28]
NA, (Cauvery basin)	Medium-Coarse	28	3.00–25.00	0	48.00–10.60	30.00–8.50	Chatterjee and Mukhopadhyay. (2002) [12]
NA, (Hongliulin coal mine)	Fine	50	4.70	0–3.63	111.12–76.47	18.84–15.15	Chen et al. (2021) [5]
Buntsandstein, (Pfinztal)	Fine	40	8.00	0	114.00–142.00	16.70–26.40	Egert et al. (2018) [29]
Buntsandstein, (Tennenbach)	Fine	40	9.00	0	42.00–47.00	11.60–11.50	
NA, (Linyi)	Coarse	50	6.00	0–5.13	60.85–29.11	NA	Geng and Cao. (2020) [6]
Buntsandstein, (France)	Medium	20	3.40–18.50	0	242.70–58.20	39.60–16.10	Heap et al. (2019) [30]
Red sandstone, (Yichang)	Medium	50	12.69	0–6.25	32.00–19.00	5.00–2.30	Huang et al. (2021) [1]
Red, Berea, and Buff sandstone, (Utah and Ohio)	Fine	55	5.60–23.00	0	183.00–75.00	25.00–11.60	Kim et al. (2017) [31]
Red sandstone, (Yunnan)	Fine	50	8.50	0, 3.3	147.3, 112	NA	Li et al. (2019) [7]
NA, (NA)	Fine	50	3.00–7.60	0	87.20–27.90	20.00–6.40	Huamin et al. (2018) [14]
Red sandstone, (Hunan)	Medium	50	5.20–5.30	0	60.73–64.62	10.53–10.36	Lin et al. (2020) [32]
Shanxi, (Huaibei and Xuzhou mining areas)	Fine	25	7.90	0–2.96	66.45–40.62	10.17–5.24	Lu et al. (2017) [33]
NA, (Atovgvia da Baleia)	Fine	50	3.60–18.60	0	135.70–17.60	NA	Ludovico-Marques et al. (2012) [15]
Gosford, (Sydney basin)	Coarse	42	18.00	0–6.90	43.98–11.69	6.58–1.98	Masoumi et al. (2017) [8]
Hawkesbury sandstone (Australia)	Medium	42	12.50	0	38.81–77.22	8.00–11.60	Roshan et al. (2018) [34]
NA, (Dholpur)	Fine	50	21.00	0	31.14–37.68	11.98–7.48	Sirdesai et al. (2018) [35]
Red sandstone, (Hunan)	Fine	50	11.60	0–3.40	108.00–55.50	16.80–11.30	Tang et al. (2018) [9]
NA, (Longchang)	Fine	50	4.70	0–1.61	127.45–61.17	20.98–12.34	Shibin Tang. (2018) [10]
NA, (Perth and Sydney basin)	Fine	38	13.00–16.00	0	65.01–32.37	13.34	Wasantha et al. (2018) [11]
Jiaozuo, (Henan)	Fine	50	5.30–16.00	0.38	140.00–54.00	34.50–6.25	Wu et al. (2013) [36]
Red sandstone, (Hongyang)	Fine	50	6.48	0	101.28–107.38	NA	Wu et al. (2018) [19]
NA, (Chongqing)	Coarse	25	8.10	0	42.40	7.23	Xu et al. (2017) [37]
NA, (Rizhao)	Fine-Medium	50	6.88	0	134.45–137.99	28.78–27.16	Sheng-Qi Yang. (2016) [38]
Red sandstone, (Hunan)	Fine	50	12.60	0–4.70	75.00–48.00	10.95–7.70	Yu et al. (2019) [4]
Red sandstone, (Ganzhou)	Fine	50	2.80	0–2.77	96.58–53.07	16.00–10.60	Zhao et al. (2021) [39]
Black sandstone, (NA)	Medium	50	1.50	0	93.64	19.47	Zhou et al. (2018) [20]
Red sandstone, (NA)	Medium	50	2.00	0	43.32	8.52	

NA—Not Available, D—Specimen diameter in mm, *n*—Porosity in percentage, *w*—water content in percentage, UCS—Uniaxial Compressive Strength in MPa, *E*—Young’s modulus in GPa.

**Table 4:** Assessment of prediction equations for UCS based on the testing dataset.

Sandstone Formation	Sandstone Location	Equation	RMSE	MAD	Reference
Table 1, Table 2 and Table 3	Wyoming and literature data	$\hat{\mathrm{UCS}}=108.46-2.23\;n\%-8.78\;w\%-38.77\;d_{m}$	31.60	22.13	This study
NA	Krishna-Godavari Basin, India	$\hat{\mathrm{UCS}}=-2.16\;n\%+52.84$	50.91	38.72	Chatterjee and Mukhopadhyay (2002) [12]
NA	Cauvery Basin, India	$\hat{\mathrm{UCS}}=-0.79\;n\%+30.88$	58.31	45.55	Chatterjee and Mukhopadhyay. (2002) [12]
NA	Shanxi Province, China	$\hat{\mathrm{UCS}}=44.6\;e^{-0.399w\%}+66.60$	46.80	37.24	Chen et al. (2021) [5]
NA	Atouguia da Baleia, Portugal	$\hat{\mathrm{UCS}}=206.7\;e^{-0.129\;n\%}$	50.55	38.64	Ludovico-Marques et al. (2012) [15]
Gosford sandstone	Sydney Basin, Australia	$\hat{\mathrm{UCS}}=43.63\;e^{-0.20w\%}$	48.50	36.60	Masoumi et al. (2017) [8]
Red sandstone	Hunan Province, China	$\hat{\mathrm{UCS}}=55.21\;e^{-0.7502w\%}+51.6$	40.63	29.82	Tang et al. (2018) [9]
Black sandstone	Sichuan Province, China	$\hat{\mathrm{UCS}}=80.604\;e^{-0.9044w\%}+43.17$	46.44	34.11	Shibin Tang. (2018) [10]
Red sandstone	Jiangxi Province, China	$\hat{\mathrm{UCS}}=70.8734\;e^{-0.3188\;w\%}+26.84$	37.01	26.59	Zhao et al. (2021) [39]

NA—Not available, UCS—Uniaxial compressive strength in MPa; *w*—water content in percentage, and *n*—rock porosity in percentage, RMSE—Root Mean Square Error, MAD—Mean Absolute Deviation.

**Table 5:** Summary of triaxial compression test results of dried sandstones from Wyoming.

Sample ID	Formation	Geological Age	Depth m	D, mm	*n*, %	$\sigma_{3},\;\mathrm{MPa}$	$\sigma_{1},\;\mathrm{MPa}$	*E*, GPa	c, MPa	ϕ, Degree
16	Flathead	Cambrian	4.76	50	2.20	4	95.59	61.33	20.34	37
			5.85	50	5.35	10	120.12	41.027		
17	Cloverly	Cretaceous	30.18	50	17.80	4	15.89	36.57	3.45	22
			30.03	50	19.50	10	31.90	27.34		
18	Sundance	Jurassic	3.35	50	22.50	8	38.58	13.91	6.07	18
19	Aspen	Cretaceous	6.10	50	3.43	10	98.89	34.31	5.38	49
20	Aspen	Cretaceous	11.52	50	5.62	4	45.85	53.52	5.52	49
			11.65	50	7.18	10	99.53	1.41		
21	Denver and Arapahoe	NA	NA	50	30.40	1	8.71	0.097	2.62	18
				50	30.00	4	15.57	1.095		
				50	31.20	10	25.92	0.21		
23	Lance	Cretaceous	Surface	50	9.31	2	5.07	1.72	11	8
				50	12.18	4	36.50	4.64		
31	Tensleep	Pennsylvanian	Surface	50	13.00	1	70.24	18.67	13.1	48
				50	13.20	4	59.88	17.25		
				50	13.11	8	120.39	8.48		
32	Arikaree	Lower Miocene	Surface	50	11.70	4	45.86	33.99	2.76	51
				50	14.00	10	93.80	NA		
33	Arikaree	Lower Miocene	Surface	50	12.20	4	46.49	8.95	3.1	54
				50	11.10	10	111.03	0.34		
39	Hanna	Paleocene	44.15	25	13.10	4	33.19	3.14	2.48	46
			44.60	25	15.20	10	70.72	5.82		
41	Hanna	Paleocene	23.32	25	14.80	4	31.47	4.09	2.34	44
			24.09	25	16.80	6	42.96	4.17		
43	Wind River	Eocene	Surface	25	14.50	4	14.00	17.90	8.96	56
				25	14.00	10	21.11	7.70		
49	Bridger	Eocene	Surface	25	27.20	4	34.68	29.59	4.48	29
				25	24.20	10	42.44	4.40		
50	Fort Union	Paleocene	Surface	25	12.20	4	31.48	3.52	1.38	47
51	Fort Union	Paleocene	Surface	25	5.92	6	89.69	5.03	5.52	55
				25	3.92	8	147.00	7.70		
56	Casper	Permian	Surface	25	11.10	4	72.56	14.38	7.93	53
				25	10.20	10	132.23	16.99		
				25	14.00	10	21.11	7.70		

NA—Not Available, D—Specimen diameter in mm, *n*—Porosity in percentage, $\sigma_{3}$—Confining stress in MPa, $\sigma_{1}$—Peak Compressive Strength in MPa, *E*—Young’s modulus in GPa, c—cohesion in MPa, ϕ—Internal friction angle.

**Table 6:** Summary of triaxial compression test results of sandstones from the literature.

Formation, (Location)	Size Classification	D, mm	*n*, %	*w*, %	$\sigma_{3},\;\mathrm{MPa}$	$\sigma_{1},\;\mathrm{MPa}$	*E*, GPa	c, MPa	ϕ, Degree	Reference
Vosges sandstone (France)	Medium	50	22.00	0	0.1–60	32.10–175.00	NA	NA	NA	Bésuelle et al. (2000) [52]
Buntsandstein, (Pfinztal)	Fine	40	8.00	0	50–90	337.00–471.00	11.80–13.00	40	35	Egert et al. (2018) [29]
Buntsandstein, (Tennenbach)	Fine	40	9.00	0	50–90	251.00–358.00	10.90–10.20	24	31	
Red sandstone, (Yichang)	Medium	50	12.69	0–6.25	10	121.00–86.00	12.00–8.45	NA	NA	Huang et al. (2021) [1]
Yellow sandstone, (Meishan)	Medium	50	21.00	0	2–8	106.12–164.23	NA	NA	NA	Huang et al. (2021) [1]
NA, (Yunnan)	Fine	50	8.50	0	2–10	110.02–185.97	26.71–30.97	13.86	56.29	Kegang et al. (2016) [53]
Red sandstone, (Yunnan)	Fine	50	8.50	0	10–40	147.00–245.00	19.00–17.00	28.07	38.38	Li et al. (2019) [7]
			8.50	3.30	10–40	112.00–175.00	16.30–15.20	25.68	32.89	
NA, (Qinghai)	Fine	50	1.63	0	1–3	120.00–127.60	NA	27.74	38.32	Liping et al. (2019) [54]
	Coarse	50	1.92	0	1–3	109.30–122.30	NA	20.74	46.32	
Hawkesbury, (Sydney basin)	Medium	50	16.00	0	10–30	109.90–172.40	NA	22.6	31.5	Roshan et al. (2017) [34]
NA, (Xiangjiaba)	Fine	50	2.64	0	3–20	144.81–266.08	27.18–31.12	32	44	Wang et al. (2020) [55]
Shanxi, (Henan)	Fine-Coarse	50	6.53	0	10–50	187.81–283.93	NA	40	32	Wang and Cui. (2018) [56]
Hawkesbury, (Sydney)	Medium	54	13.00	0	4–25	58.00–116.00	NA	16.5	28	Wasantha and Ranjith. (2014) [57]
NA, (Chongqing)	Coarse	25	8.10	0	5–40	77.00–219.09	8.03–16.07	30.16	38.4	Xu et al. (2017) [37]
NA, (Rizhaou)	Fine-Medium	50	6.88	0	8–35	198.92–316.38	29.03–33.69	30.58	45.7	Sheng-Qi Yang. (2016) [38]
Red sandstone, (Shandong)	Fine-Medium	55	6.48	0	5–35	115.10–242.90	18.81–23.80	22.42	37.8	Yang and Jing. (2013) [58]
Black sandstone, (NA)	Medium	50	1.50	0	10–60	131.86–241.81	19.89–21.71	22.5	33.5	Zhou et al. (2018) [20]
Red sandstone, (NA)	Medium	50	2.00	0	10–60	93.40–131.49	10.76–9.90	13	23	

NA—Not Available, D—Specimen diameter in mm, *n*—Porosity in percentage, *w*—water content in percentage, $\sigma_{3}$—Confining stress in MPa, $\sigma_{1}$—Compressive Strength in MPa, *E*—Young’s modulus in GPa, c—cohesion in MPa, ϕ—Internal friction angle in degree.

**Table 7:** Assessment of prediction equations based on the testing dataset.

Sandstone Formation	Sandstone Location	Equation	Reference	RMSE	MAD
Table 5 and Table 6	Wyoming and literature data	$\hat{\sigma_{1}}=90.68+1.63\;n\%+2.9\;\sigma_{3}-0.12\;n{\%}^{2}+0.0042\;\sigma_{3}^{2}$	This study	50.19	40.60
General	General	$\hat{\sigma_{1}}=\sigma_{3}+\sigma_{c}\;\sqrt{m_{i}\times \frac{\sigma_{3}}{\sigma_{c}}+1}$	Generalized Hoek and Brown criterion, 1980 [25]	62.62	41.64
NA	Linyi	$\hat{\sigma_{1}}=9.008\;\sigma_{3}+83.56$	Gong et al., 2019 [59]	169.05	119.59
Red Sandstone	Shandong	$\hat{\sigma_{1}}=3.9766\;\sigma_{3}+109.1850$	Wu et al., 2018 [19]	62.15	45.51
Yellow Sandstone	Zunyi	$\hat{\sigma_{1}}=4.36\;\sigma_{3}+77.33$	Yang et al., 2020 [60]	57.18	44.40

NA—Not Available, $\sigma_{1}$—Compressive strength in MPa, $\sigma_{3}$—Confining stress in MPa, *n*—Porosity in percentage, $\sigma_{c}$—Unconfined compressive strength, $m_{i}$—Material constant, RMSE—Root mean square error, MAD—Mean Absolute Deviation.

**Table 8:** Assessment of prediction equations based on the testing dataset.

Sandstone Formation	Sandstone Location	Equation	Reference	RMSE	MAD
Table 1 and Table 4	Wyoming and literature data	$\hat{E}=0.17\;\mathrm{UCS}$	This study	9.79	7.05
Krishna-Godavari and Cauvery basin	India	$\hat{E}=0.73\;\mathrm{UCS}+0.17$	Chatterjee and Mukhopadhyay. (2002) [12]	33.35	26.90
Upper Silesia Basin	Poland	$\hat{E}=0.17\;\mathrm{UCS}+2.907$	Malkowski et al. (2018) [61]	9.81	8.13
Island Creek	US Bureau of mines	$\hat{E}=0.05\;\mathrm{UCS}+20.6$	Rohde and Feng. (1990) [62]	12.17	10.59

RMSE—Root mean square error, MAD—Mean Absolute Deviation, UCS—Uniaxial compressive strength in MPa, *E*—Young’s modulus in GPa.

## Data Availability

The data presented in this study are available on request from the corresponding author. The data are not publicly available due to unpublished technical report to the funding agencies.

## Copyright Statement

The State of Wyoming, WYDOT, and the University of Wyoming reserve a royalty-free, nonexclusive, unlimited, and irrevocable license to reproduce, published, or otherwise use, and authorize others to use the copyright in any work that is generated from the research project entitled Improving Design and Construction of Transportation Infrastructure through Bedrock Characterization.
